# Mechanisms and prevention of thermal injury from gamma radiosurgery headframes during 3T MR imaging

**DOI:** 10.1120/jacmp.v13i4.3613

**Published:** 2012-07-05

**Authors:** Marcus C. Bennett, David B. Wiant, Jacob A. Gersh, Wendy Dolesh, X. Ding, Ryan C. M. Best, J. D. Bourland

**Affiliations:** ^1^ Department of Radiation Oncology Wake Forest University School of Medicine Winston‐Salem NC USA; ^2^ Department of Biomedical Engineering Wake Forest University School of Medicine Winston‐Salem NC USA; ^3^ Department of Physics Wake Forest University School of Medicine Winston‐Salem NC USA

**Keywords:** MRI‐induced heating, radiosurgery, stereotactic headframe

## Abstract

Magnetic resonance imaging (MRI) is regularly used for stereotactic imaging of Gamma Knife (GK) radiosurgery patients for GK treatment planning. MRI‐induced thermal injuries have occurred and been reported for GK patients with attached metallic headframes. Depending on the specific MR imaging and headframe conditions, a skin injury from MRI‐induced heating can potentially occur where the four headframe screws contact the skin surface of the patient's head. Higher MR field strength has a greater heating potential. Two primary heating mechanisms, electromagnetic induction and the antenna effect, are possible. In this study, MRI‐induced heating from a 3T clinical MRI scanner was investigated for stereotactic headframes used in gamma radiosurgery and neurosurgery. Using melons as head phantoms, optical thermometers were used to characterize the temperature profile at various points of the melon headframe composite as a function of two 3T MR pulse sequence protocols. Different combinations of GK radiosurgery headframe post and screw designs were tested to determine best and worst combinations for MRI‐induced heating. Temperature increases were measured for all pulse sequences tested, indicating that the potential exists for MRI‐induced skin heating and burns at the headframe attachment site. This heating originates with electromagnetic induction caused by the RF fields inducing current in a loop formed by the headframe, mounting screws, and the region of the patient's head located between any of the two screws. This induced current is then resistively dissipated, with the regions of highest resistance, located at the headframe screw–patient head interface, experiencing the most heating. Significant heating can be prevented by replacing the metallic threads holding the screw with electrically insulated nuts, which is the heating prevention and patient safety recommendation of the GK manufacturer. Our results confirm that the manufacturer's recommendation to use insulating nuts reduces the induced currents in the headframe nearly to zero, effectively preventing heating and minimizing the likelihood of thermal injury.

PACS numbers: 87.57.‐s, 87.61.‐c, 87.61.Tg, 87.57.c‐

## I. INTRODUCTION & BACKGROUND

Magnetic resonance imaging (MRI) is increasingly used in radiation oncology departments for radiation treatment planning because of the excellent contrast for soft tissues and tumors.^(^ ^1^ ^–^ ^3^ ^)^ MRI is also considered a primary imaging modality for Gamma Knife (GK) stereotactic radiosurgery treatment planning. Important MRI safety issues that may be new to the radiation oncology clinic include potential damage and injury to property, patients and staff from several sources: the rapid acceleration of nearby ferromagnetic objects by the high‐static magnetic field (up to 3 T for clinical units);^(^ ^4^ ^–^ ^11^ ^)^ the gradient fields, which have been shown to induce nerve stimulation in humans;^(^ ^12^ ^,^ ^13^ ^)^ the cryogenics, which can cause severe frostbite, suffocation, and substantial explosions if the pressure relief system of the cryogen containers become defective;^(^ ^14^ ^)^ and the radiofrequency (RF) fields, which are likely the primary source of MRI‐induced thermal injury.^(^ ^9^ ^,^ ^15^ ^–^ ^23^ ^)^

This study is motivated by the increasing number of reports of MRI‐induced patient thermal injuries, including burns,^(^ ^9^ ^,^ ^13^ ^–^ ^23^ ^)^ and by our clinic's use of a dedicated 3T MR simulator^(^ ^1^ ^,^ ^2^ ^)^ that serves as an integral part of an active GK radiosurgery program. The causes of reported MRI‐induced thermal injuries and burns are often not well understood, are sometimes described as unknown or mysterious, and seem to originate with different heating mechanisms. In some cases, burns were associated with wires used with electronic monitoring equipment or implanted biomedical devices such as pacemakers,^(^ ^8^ ^–^ ^11^ ^,^ ^15^ ^,^ ^17^ ^,^ ^20^ ^–^ ^24^ ^)^ while in other cases, thermal injuries have occurred with no wires present near the patient, in the extremities or around tattoos,^(^ ^24^ ^)^ for instance. While the specific situations leading to these injuries may be difficult to pinpoint, the heating mechanisms causing them are not mysterious. They are the well‐understood physical phenomena of electromagnetic induction and the antenna effect, both originating with the radiofrequency (RF) outputs of the MRI machine.

In 2003, headframe and GK manufacturer, Elekta, (Elekta AB, Stockholm, Sweden) notified users of the availability of “insulated posts”, with the stated use for “high tesla MR units and high frequency MR sequences”.^(^ ^25^ ^)^ Beginning at approximately the same time, reports to the US Food and Drug Administration (FDA) documented thermal injuries due to MR‐induced heating for patients wearing stereotactic headframes.^(^ ^26^ ^–^ ^33^ ^)^

The physical explanation of these reported thermal injuries has not been given. Thus, this study seeks to prevent MRI‐induced burns in GK patients by first understanding the physical mechanisms that could lead to these injuries and, subsequently, validating the technique recommended to prevent them. The manufacturer's recommended burn prevention technique is to replace the tapped holes at the GK headframe screw–post interface, a metal‐to‐metal junction, with snap‐in insulated nuts. The use of the insulated headframe posts is required for both 1.5 T and 3 T MRI scans.^(^ ^34^ ^)^ The use of uninsulated posts is permitted for X‐ray‐only procedures, such as CT scans or biplanar projection angiography.

### A. MRI‐induced heating mechanisms

Previous studies of MRI‐induced burns considered three potential heating mechanisms:^(^ ^15^ ^,^ ^16^ ^)^ 1) resistive heating from currents induced by direct electromagnetic induction, 2) the unlikely coincidental presence of a conducting loop arranged perpendicular to the RF field and containing the right combination of inductance and capacitance to result in a resonant frequency equal to that of the MRI RF field (a special case of electromagnetic induction), and 3) the antenna effect, which is antinodal heating at the tips of wires or other conductors of appropriate length that act as antennas. A brief review of these potential heating mechanisms is presented.

The first mechanism, electromagnetic induction, is described by Faraday's law:

(1) $$\oint \vec{E}\cdot \vec{dl}=-d{/}_{dt}(\int \vec{B}\cdot d\vec{a})$$

where $\bar{E}$ is the electric field, $\bar{l}$ is the distance around the loop, $\bar{B}$ is the magnetic flux density, and $\bar{a}$ is the cross‐sectional area enclosed by a conducting loop. This can be stated more simply as:

(2) V∝dB/dt

where *V* is the voltage induced in the loop, *B* is the magnetic field, and *t* is time. In this case, the rapidly changing magnetic fields induce a current in loops of wire or other conducting material, with the area enclosed by the loop oriented perpendicular to the changing magnetic field. The voltage in turn induces a current:

(3) i=V/R

where *V* is the voltage and *R* is the resistance of the loop. The induced current is then dissipated as heat at a rate:

(4) $$P=i^{2}R$$

with the greatest heating occurring at locations with the highest resistance. These loops can be formed by wires, other conducting material such as the GK headframe posts, and/or loops of human tissue such as a patient with his arm forming a closed loop, or human tissue plus a section of wire forming a loop. The current will dissipate via resistive heating, the majority of which will occur at the position of highest electrical resistance which tends to occur at the skin–skin interface (e.g., loop formed by the arm), or the wire–skin interface (e.g., loop formed by a wire).

In the study by Dempsey et al.^(^ ^16^ ^)^ different diameter loops of copper wire were placed perpendicular to the RF field and the temperature rise was recorded. Larger diameter loops resulted in more heating, consistent with Faraday's law. No resistor was placed in the loop and, therefore, the resistive heating was distributed evenly around the loop, resulting in a temperature rise along the whole loop. Had a resistor been placed in the loop, most of the heat would have been dissipated locally at and near the resistor, resulting in a higher temperature rise over a much smaller region of the loop. Whether the induced currents originated with the RF field or with the gradient field was not resolved in the Dempsey study. However, direct measurement of the induced voltage waveform in a loop of wire showed that the source of the heating is primarily the RF field.^(^ ^17^ ^)^ Subsequent measurements confirmed this result, showing that reducing the magnitude of the RF signal reduces the induced voltage in the loop of wire.^(^ ^17^ ^)^

Resonance heating is a second possible heating mechanism. Dempsey et al.^(^ ^16^ ^)^ found very high temperature rises of up to 61°C in loops with appropriately valued inductance and capacitance to cause resonance. The resonant frequency of the loop is given by:

(5) $$f=\frac{1}{2\pi \sqrt{\text{LC}}}$$

In practice, it is unlikely that the loops described above will by coincidence happen to have the appropriate values of inductance (L) and capacitance (C) so that the resonant frequency matches the frequency of the MRI machine; however, if they do, there will be substantial heating.

The third mechanism that can lead to heating is the antenna effect, which occurs when a wire of appropriate length is exposed to the RF frequency and acts as an antenna. This effect is exploited in half‐wavelength dipole antennas for receiving radio signals. If a length of wire or other conductor is ~λ/2, an electromagnetic oscillation (resonance) will be produced with a node in the center of the wire and an antinode at either end. The maximum amplitude will occur at the antinodes, resulting in the ends of the wire heating to high enough temperatures for thermal injury. Dempsey and Condon^(^ ^15^ ^)^ showed that for a 1.5 T MRI machine with operating frequency of 63.87 MHz, the antenna effect occurred in a wire length of ~220 cm, in rough agreement with the theoretical expectation of λ/2 approximately equal to 235 cm. Local temperature increases of up to 63°C were observed at the wire tip, high enough to cause burns to the experimental apparatus,^(^ ^16^ ^)^ and certainly high enough to cause severe or ablative tissue injury. This mechanism is suspected in a number of cases of MRI induced patient burns, including pulse oximeter wires.^(^ ^24^ ^)^ It is extremely important to realize that the length of an ideal λ/2 antenna inside the human body is reduced by an order of magnitude, because the EM wavelength is significantly reduced due to the dielectric and electrical properties of human tissues. For instance, pacemaker leads in human‐equivalent soft tissue will resonate at lengths of ~20 cm for 1.5 T (64 MHz), or ~10 cm for 3 T,^(^ ^20^ ^)^ not the 220 to 235 cm in‐air values reported earlier.^(^ ^15^ ^)^

The antenna effect in conductive tissues is explained as follows. The human body is conducting, and thus the behavior of incident EM waves is described by Maxwell's equations applied to conducting media. For this case, Ampere's law is:

(6) $$\vec{\nabla }\times \vec{B}=\mu \varepsilon^{d\vec{E}}{/}_{dt}+\mu \sigma \vec{E}$$

where $\bar{B}$ is the magnetic field, $\bar{E}$ is the electric field, μ is the permeability, ɛ is the permittivity, and σ is the conductivity. The solutions to the resulting wave equations are plane waves, but with a complex wave number

(7) $$\tilde{k}=k+\mathrm{iK}$$

where

(8) $$k=\omega \sqrt{\varepsilon \mu {/}_{2}}{\left[\sqrt{1+{\left(\sigma {/}_{\varepsilon \omega }\right)}^{2}+1}\right]}^{1{/}_{2}}$$

and

(9) $$K=\omega \sqrt{\varepsilon \mu {/}_{2}}{\left[\sqrt{1+{\left(\sigma {/}_{\varepsilon \omega }\right)}^{2}-1}\right]}^{1{/}_{2}}$$

The wavelength, λ, in the human body is thus given by:

(10) $$\lambda =\frac{1}{k}$$

which depends primarily on the permittivity, ɛ, the conductivity, σ, and the frequency, ω. Another result is that the amplitude of the wave will decrease with increasing penetration depth into the human body, quantified by the skin depth, δ

(11) $$\delta =\frac{1}{K}$$

which describes the depth at which the amplitude is decreased by 1/e, or to about 37% of the surface value. For given ω, the wavelength depends on ɛ, and σ, and the wave amplitude depends on its depth below the surface, quantified by the skin depth. Accordingly, the behavior of EM waves in the human body varies within the different organs and tissue types, based on ɛ and σ values over the frequency range.^(^ ^35^ ^,^ ^36^ ^)^ It is also important to note that antenna effect heating depends on the angle of the conductor with respect to the applied EM wave and is maximized when the length of the conductor is parallel to the direction of the EM wave.

The antenna effect for metallic implants has been studied in tissue equivalent body^(^ ^20^ ^)^ and head^(^ ^35^ ^,^ ^36^ ^)^ phantoms (e.g., matched ɛ and σ). For the brain, for 3 T, the wavelength is estimated to be 25.5 cm,^(^ ^35^ ^,^ ^36^ ^)^ suggesting that an implant length of ~12.75 cm would be susceptible to antenna effect heating. At 7 T, wavelength becomes 10.6 cm leading to potential antenna effect heating for 5.3 cm implants.^(^ ^35^ ^,^ ^36^ ^)^ These implant dimensions give an indication of possible conductor lengths of relevance for antenna effect heating for GK patients wearing headframes.

All reported MRI‐induced burns likely originate in some form from either electromagnetic induction or the antenna effect. For the case of the headframe screw heating, the results of this study show that the cause is electromagnetic induction. This finding is consistent with the dimensions of the GK headframe which, with an effective unwrapped length of ~62 cm, is too short for antenna effect heating at 3 T (128 MHz, λ/2 approximately equal to 117 cm) or 1.5 T (64 MHz, λ/2 approximately equal to 220 cm).^(^ ^16^ ^)^ Also, the typical ~5−10 mm length of the screws embedded in the head surface is less than the ~13 cm length expected for the antenna effect in the human head to occur. Thus, the cause of heating is most likely due to the induction of currents in loops formed by the headframe and the tissue of the patient's head, with the area of the loop oriented perpendicularly to the rapidly changing magnetic fields. Because the heat source is resistive heating, $P=i^{2}R$ (Eq. (4)), one needs only to increase the resistance of the part of the loop outside the patient's head (i.e., the headframe) in order to decrease the resistive heating in the tissue.

The following experimental results using melon phantoms show that heating occurs during standard MR brain scans near attached GK headframe screws, the heating mechanism is electromagnetic induction, the amount of heating depends on the material type of the headframe screws and posts, and the use of insulated headframe posts renders the induced currents and associated resistive heating negligible.

## II. MATERIALS AND METHODS

### A. Experimental geometry, pulse sequences, and temperature measurements

**Figure 1:**
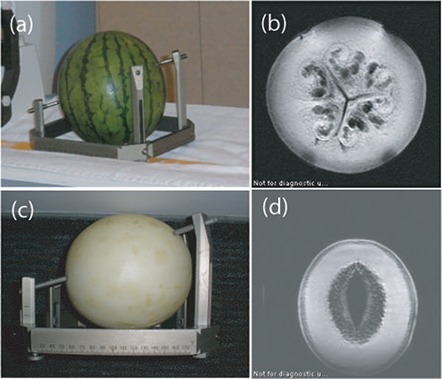
Photograph and associated MRI images of a watermelon (a) and (b), and a honeydew melon ((c) and (d)). Head‐frame screws should be kept in the shell region as indicated in the MRI images in order to maintain resistance in the MΩ range and thus be comparable to the human head.

**Figure 2:**
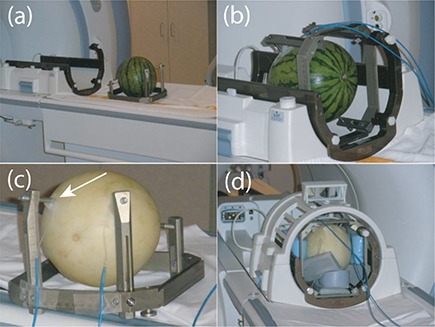
Watermelon mounted in headframe (a); watermelon placed in GK head coil (b) with optical thermometers taped to the surface of the watermelon up against the screw–melon surface interface; a similar set‐up for a honeydew melon (c). It is important to cushion the melon in the head coil (d) to reduce the vibration, which can lead to thermometers coming loose from the surface.

Fresh watermelons and honeydew melons were used to simulate the human head (Fig. 1). The melons ranged in weight approximately from 1.5 to 3 lbs. The electrical resistance of the surface layer of both types of melon, about 1 MΩ, is comparable to the resistance of the human head measured by point contacts on the skin. Both displayed similar resistive properties, with R~1 MΩ within the outer shell of the melon, but decreasing to ~50−300kΩ if the inner pulp is penetrated by the meter lead (Fig. 1). As shown in Fig. 2, the melons are mounted in the GK headframe and optical thermometers are mounted with tape at various positions on the headframe, screws, and melon. All experiments were conducted using a GE 3.0 T MR scanner (Signa EXCITE, GE Healthcare, Waukesha, WI). Two pulse sequences were investigated: 1) a standard T1‐weighted GK Protocol sequence (axial T1 spin echo, with flow compensation, TE=~23 ms, TR=800 ms, NEX=1, BW=22.73, and a 384× 224 matrix), and 2) in order to induce increased heating, an enhanced 13 minute fast spin echo sequence (FSCXL with flow compensation and tailored RF, TE=16 ms, TR=767 ms, NEX=4 MS, BW=20.83, ETL=37, and a 256× 256 matrix). Temperature was measured using two MR‐compatible fiber optic thermometers, (Veris MR Vital Signs Monitoring System, Medrad, Inc, Warrendale, PA). The temperature is determined via a temperature sensitive phosphor located at the probe tip and energized by an LED pulse.^(^ ^37^ ^)^ Thermal connections to the headframe screws were made by careful adhesion of the more thermally conductive *side* (as opposed to the end) of the optical thermometer to the tip of the screw (arrow, Fig. 2(c). Thermal connections to the melon were made by enclosing the optical thermometers in thin plastic wrap and inserting them directly into the melon to a depth of 5 mm, preventing air cavities and ensuring good thermal contact.

### B. Antenna‐effect heating

**Figure 3:**
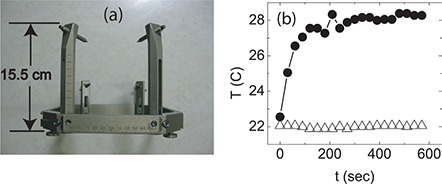
Photograph of Gamma Knife headframe (a) showing that the length of the longest post is ~15 cm, which is much lower than the expected length required for heating via the antenna effect, which would be ~60 cm. Plot (b) of the time dependence of the temperature taken at the top of the headframe post, showing that there is no heating with the melon absent (triangles), but significant heating at the screw tips when the melon is mounted in the headframe (circles).

To verify the absence of antenna‐effect heating, the GK headframe alone with 4 uninsulated posts and 4 tungsten tipped alumina screws (Fig. 3(a)) was placed in the GK standard four‐element head coil and scanned in the MRI using the current standard T1‐weighted GK protocol pulse sequence. Temperature measurements at the two anterior headframe screws were recorded every 30 seconds during the 9.5 minute pulse sequence. No heating was detected at either screw, confirming that the headframe–post–screw unit was not behaving as an antenna (Fig. 3(b)), triangles). Similarly, 45 and 60 mm titanium screws alone, without the headframe or posts, were embedded in the melon to depths of 5 mm and evaluated for heating with the T1‐weighted pulse sequence. Again, no heating was observed at the screw–melon interface, indicating the absence of the antenna effect for these length screws for the 0.5 mm portion of the screw within the melon.

### C. Electromagnetic induction heating

**Figure 4:**
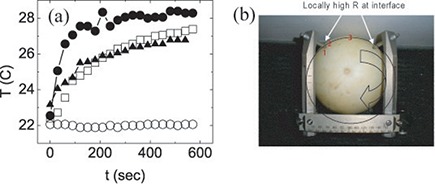
The time dependence (a) of the temperature measured at each of the three positions indicated in (b) (filled triangles, hollow squares, hollow circles) and on the screw surface near the screw tip (filled circles). Photograph (b) of a honeydew melon showing three locations (red numbers 1–3) where the time dependence of the temperature was measured during a test scan. The arrow indicates the approximately circular current path of the induced current.

To determine whether there is any heating from electromagnetic induction in a loop, a water melon was subsequently mounted on the same headframe and remeasured in the same four‐element head coil using the same pulse sequence. The screws were screwed approximately 5 mm into the surface of the watermelon and the temperature was again measured every 30 seconds at the top part of the anterior screw tips at the melon surface. A temperature increase of approximately 6°C near the screw tips was observed, confirming electromagnetic induction as the source of the heating (Fig. 3(b)), circles). To further characterize the temperature profile of the melon headframe composite, a watermelon was mounted on the GK headframe and with the T1 axial GK pulse sequence, the temperature was measured at the screw tip and three positions along the melon in order to determine where heating occurs. Heating was greatest along the screw surface and at two points in the melon nearest to the screw (Fig. 4).

### D. Screw–post combinations

**Figure 5:**
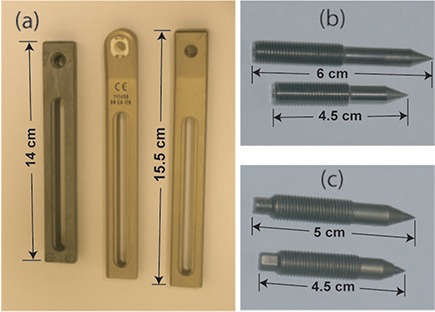
The three types of headframe post (a) that were used in the experiment were from left to right, carbon, alumina with the snap in insulated threads, and uninsulated alumina; titanium screws (b); tungsten‐tipped alumina screws (c).

With the heating mechanism established as electromagnetic induction, the heating characteristics of different screw and post types were measured. The temperature profiles were measured for combinations of two different screw types, and two different headframe post materials. All screw and post comparison tests were done on the same melon in immediate succession to ensure the same measurement conditions. Baseline temperature was determined by the initial temperature measured just before starting the initial scan, and subsequent scans were started only after temperatures cooled to within 1°C of baseline. Baseline temperatures varied from day to day from 18°C to 23°C, with a typical value of 20°C. Titanium‐ or tungsten‐tipped alumina screws were measured in combination with either carbon or alumina posts, with both regular threads and insulated (plastic) nuts (Fig. 5). These combinations were chosen to represent the possible configurations available in the clinic, as well as to represent a range of safety (from “not safe” through “safe”) (Table 1). To determine whether the manufacturer‐recommended insulating posts prevent heating, the standard alumina headframe posts were replaced with electrically insulated posts, designed by placing snap–in insulating nuts between the screw and the post in the headframe in place of the regular threaded hole (Fig. 5(a)), center). Experimental results confirm that this increased impedance prevents significant heating at the headframe screw–patient head interface. As shown in Table 1, the insulated posts are the only ones that rendered the heating negligible.

**Table 1:** General heating characteristics for different screw‐post combinations. Based on temperature profile plots (see associated figures), combinations that showed anything more than negligible heating were deemed “not safe”.

*Screw Type*	*Alumina*	*Post Type Carbon*	*Insulated Alumina*
Alumina	not safe (Fig. 11) significant heating	not safe (Fig. 12) some heating	safe (Fig. 14) negligible heating
Titanium	not safe (Fig. 11) most heating	not safe (Fig. 12) some heating	safe (Fig. 14) negligible heating

## III. RESULTS

### A. Electromagnetic induction responsible for MRI‐induced heating

The initial experiment was designed to determine which of the two most likely heating mechanisms, the antenna effect or electromagnetic induction, is responsible for the heating that occurs at the GK headframe screw–melon interface. Antenna‐effect heating can be theoretically ruled out because the dimensions of the GK headframe are not large enough for this type of heating to occur. For the 128 MHz RF field of the 3 T magnet, the RF wavelength is ~235 cm, which corresponds to λ/2~117 cm. The longest posts on the GK headframe are ~15 cm and the overall unwrapped length is ~62 cm, much shorter than the required ~117 cm necessary for antenna heating to occur in air. Antenna‐effect heating was experimentally ruled out based on temperature measurements that showed: 1) no heating for the assembled headframe suspended in air, and 2 no heating for screws of various lengths embedded at depths of 0.5 cm in the melon. This latter null result is expected based on the nominal EM wavelength produced by 3 T scans for soft tissue (~40 cm) and for brain tissue (~25 cm).^(^ ^35^ ^,^ ^36^ ^)^

Resonance‐induced heating is a remaining phenomenon that could possibly result in heating. We have measured negligible capacitances and inductances in the GK headframe–melon composite and there is no evidence of resonance heating occurring in this experiment.

### B. Induced heating location

**Figure 6:**
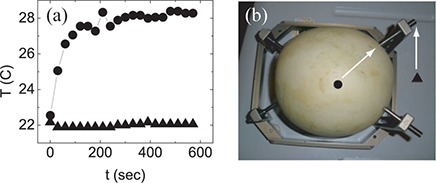
Plot (a) of the time dependence of the temperature measured near the screw tip (filled circles) and at the back part of the screw (filled triangles), as indicated in the photograph of the melon headframe composite (b).

Temperature measurements versus time as a function of position between the two anterior screws show that heating is greatest at the tapered part or tip of the screw along the melon–screw interface and that heating decreases with increasing distance from the screw tip (Fig. 4). Specifically, we found that heating occurs only near the tip of the screw and not primarily at back of the screw (Fig. 6). This temperature profile is consistent with the formation of a loop with most of the heating occurring at the maximum resistance spot in the loop, which is at the screw–melon interface in the conventional frame setup.

**Figure 7:**
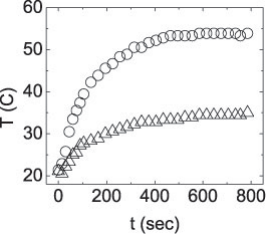
The time dependence of the temperature at the melon–screw interface for the screws penetrating the standard 5 mm depth (circles) and a much deeper penetration of approximately 2 cm (triangles). The lower resistance of the pulp of the melon located > 1 cm below the melon surface results in less heat dissipated at deeper positions.

**Figure 8:**
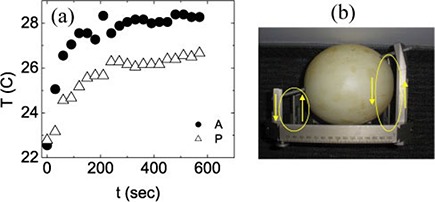
The time dependence of the temperature measured near the screw tips for the larger anterior loop (circles) and the smaller posterior loop (triangles), showing that there is more heating induced in the larger loop, as noted in (b), consistent with electromagnetic inductive heating.

**Figure 9:**
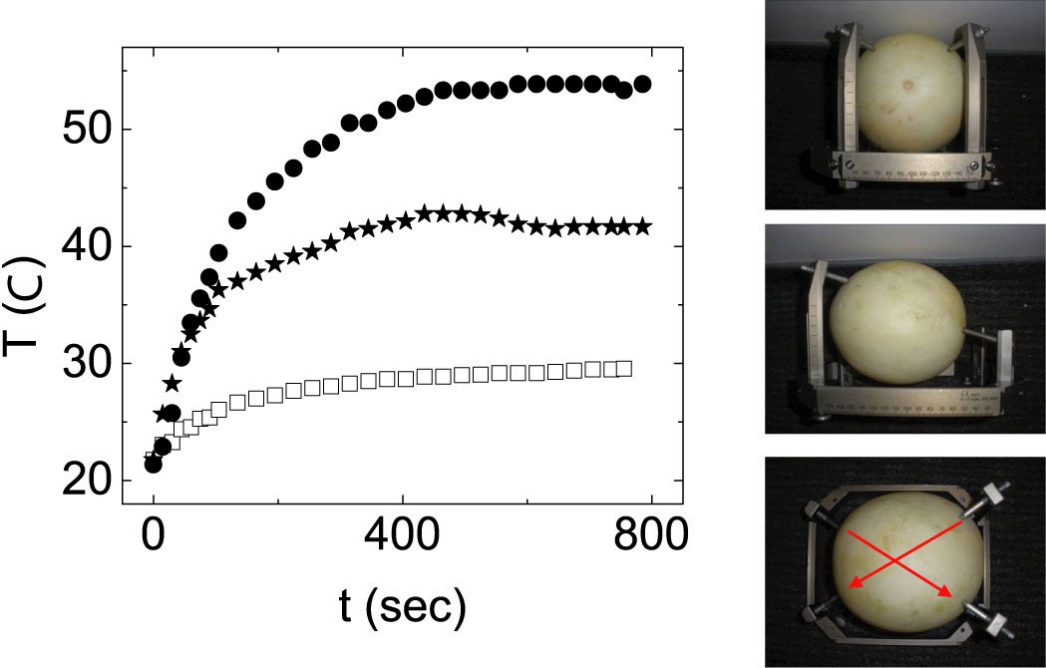
The time dependence of the temperature measured with just two headframe screws attached in the configurations depicted in the photographs, isolating the anterior loop (top, filled circles), the right loop (middle, filled stars), and a diagonal loop (bottom, hollow squares).

Melon resistivity was measured at different locations on the surface and at depth to determine variability that would impact temperature measurements. Using a handheld resistance meter, we measured a resistance of 1–3 MΩ for the outer flesh of the melon. Deeper into the melon, however, where the pulp is located, there is a significant drop in resistance down to ~100 kΩ. Experiments repeated with the thermometers placed in this deeper region show significantly decreased heating, owing to the reduced resistance (Fig. 7). This result shows that the region of heat is resistance dependent, again confirming that the source of the heating is the induced current loop with the same current heating more in higher resistance regions. To further characterize the heating that occurs in the melon headframe composite, the time dependence of the temperature was measured on the posterior loop, which would be expected to have less heating than the anterior loop due to the smaller area enclosed. In Figure 8, the time dependence of the temperature of the posterior loop (Fig. 8(a)), triangles) is plotted with that of the anterior loop (circles). As expected from current induced in accordance to Faraday's law, the induced current and its associated heating is less in the posterior loop because of the smaller cross sectional area. Other potential loops are separately measured to find the relative contributions to the screw heating of the composite system. Fig. 9 shows the time dependence of the temperature taken near the tip of the right anterior headframe screw at the screw–melon interface during the 13 minute FSE scan for each of the indicated loops. The anterior loop shows the most heating, with the side and diagonal loops each showing less heating (Fig. 9).

### C. Screw–post combinations

**Figure 10:**
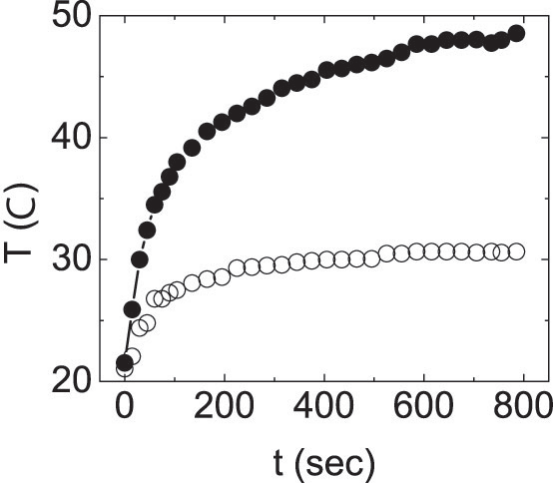
The time dependence of the temperature of the headframe screw at the screw–melon interface near the screw tip for titanium screws (filled circles) and tungsten‐tipped alumina screws (hollow circles), taken during the same pulse sequence.

**Figure 11:**
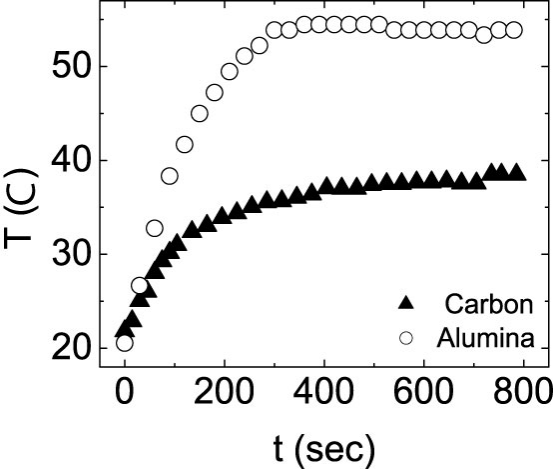
The time dependence of the temperature measured at screw tip screwed into an alumina post (circles) and a carbon post (triangles), showing that more heating occurs at the screw tip in the alumina post compared with the carbon post.

**Figure 12:**
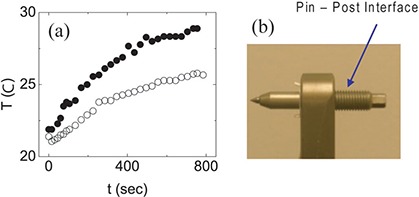
Plot (a) of the time dependence of the temperature at the threaded region of the screw–post interface as indicated in (b), showing that there is more heating at this position with the carbon post than with the titanium post, which is consistent with Faraday's law.

Temperature measurements for the two different screw materials, tungsten‐tipped alumina and titanium, show that there is more heating at the screw–melon interface for the titanium screws (Fig. 10, filled circles), indicating a higher resistance at this interface for the titanium screws compared with the alumina screws (hollow circles). Different combinations of headframe screw and post materials show different heating characteristics associated with different resistance characteristics. Several different screw–post combinations were measured. First, using the titanium screws, the time dependence of the temperature was again measured, but with carbon posts instead of alumina posts (Fig. 11). At the screw tips, the heating decreased for the carbon posts relative to the alumina post, but the heating increased at the screw–post interface (Fig. 12). This effect is also consistent with heating by electromagnetic induction. With the alumina posts, there is very little resistance at the screw–post interface, but with the carbon posts, there is significant heating, showing that there is a significant resistance at the post–screw interface. Because the size of the loop is the same, the same voltage is induced in the loop. However, now there are two locations of significant resistance, so the heat is dissipated at two locations instead of just one. Thus the heating at the screw–melon interface decreases, but increases at the screw–post interface. This occurs only for the carbon posts. Because carbon posts may offer some advantages for CT imaging, it would be possible to use these posts with minimal heating via a similar insulated nut setup as is currently used with the alumina posts.

**Figure 13:**
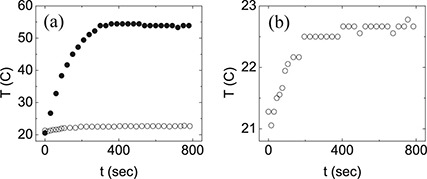
The time dependence (a) of the temperature near the screw tip for the uninsulated alumina post and titanium screws (filled circles) and for the insulated alumina post with titanium screws (hollow circles), showing the prevention of significant heating by the increased resistance of the insulating nuts. Plot (b) of the time dependence of the temperature using the insulated post showing that while the heating is almost completely eliminated, there still is some heating present, which is consistent with heating by electromagnetic induction.

**Figure 14:**
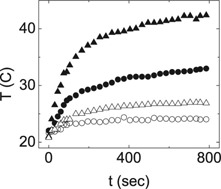
Heating from mixed‐post configurations, measured at the screw–melon interface. Circles show heating for carbon posts, with one insulated post (right anterior, hollow circles) and three uninsulated posts (temperatures obtained at left anterior, filled circles) at the other locations. Triangles show heating for alumina posts, with one insulated alumina post (right anterior, hollow triangles) and three uninsulated posts (temperatures obtained at left anterior, filled triangles) at the other locations. Uninsulated posts show higher temperature increases than insulated posts, and alumina posts have higher heating than carbon posts.

Temperature measurements with the uninsulated alumina posts replaced with the insulated posts using snap‐in insulated nuts prove that the insulated posts diminish heating to a negligible level (Fig. 13). A close–up view shows the small amount of heating (Fig. 13(b)). For mixed headframe–post–screw combinations, the results for one insulated alumina post and three uninsulated alumina posts are compared with the case for one insulated alumina post and three carbon posts (Fig. 14). As expected, there is less heating at the screw tips when carbon posts are used, compared with alumina posts.

In general, for the case of the GK headframe, the greatest heating occurs at the headframe screw–patient head interface, with the loop formed by the frame in contact with the skin. The results of this study show that this patient–frame loop is the only mechanism that can lead to GK patient burns that occur in the vicinity of the headframe screws.

## IV. DISCUSSION

This set of experiments was designed to determine which physical mechanism could lead to local heating of the GK headframe for patients undergoing stereotactic MR imaging, and to test the manufacturer's recommended method for heating prevention using electrically insulated posts. Additional experiments were conducted to characterize typical heating profiles of the GK headframe–patient system that occurs during MRI scans, and to learn how this profile changes when headframe post and screw materials are changed.

Initial experiments (Fig. 3) ruled out the antenna effect as a headframe heating mechanism and confirmed that the observed heating originates with the resistive dissipation of currents induced by the rapidly changing magnetic fields passing perpendicularly through the area enclosed by electrically conducting loops comprised of the headframe, headframe screws, and region of the patient's head between the two headframe screws, as described by Faraday's law. Further experiments showed that the resistive heating occurs primarily at the screw tips (Fig. 4, Fig. 6), where the electrical resistance is highest. Subsequent experiments found no antenna‐effect heating in the portion of the screw embedded in the melon with no headframe attached.

The heating profile can be controlled by changing the materials of the headframe posts and screws. This was observed in the experiments comparing combinations of alumina, insulated alumina, and carbon posts, and titanium‐ and tungsten‐tipped alumina screws (Figs. 10–14). Figures 11 and 12 compare the heating characteristics of the headframe composite with alumina screws and alumina posts with that of same setup, but with carbon posts in place of the alumina posts. While additional heating occurs at the carbon post–headframe screw interface (Fig. 12), causing the region of the post surrounding the headframe screw to heat, there is slightly less heating at the screw–patient head interface (Fig. 11), which again is consistent with electromagnetic induction heating. The carbon post–screw interface has a significantly higher impedance compared with the alumina post–screw interface and, thus, dissipates a larger amount of the induced current here, resulting in a reduced dissipation at the patient head–screw interface.

Replacing the uninsulated posts with the insulated posts prevented all but negligible heating of the melon headframe composite (Fig. 13), which is consistent with Faraday's law. The insulating nuts greatly increase the resistance of the loop. If, for example, the resistance of the loop is doubled, then the induced voltage, which is a function only of the cross‐sectional area of the loop, is still the same, resulting in the current being halved. Resistive heating $P=i^{2}R$, so that while the resistance is doubled, the induced current squared is quartered; therefore, the heating is halved. Thus increasing the resistance in any given loop reduces the heating when the mechanism is Faraday's law (e.g., electromagnetic induction). The manufacturer's recommended method works because the heating mechanism is electromagnetic induction. It is important to note that all four posts should be insulated, because as shown in Fig. 9, multiple loops contribute to the heating at any given screw tip. This same antiheating technique is effective for any headframe post material, and because carbon posts may offer some advantages in CT scans, it would be possible to use these posts with minimal heating by using the same insulated nut setup that is currently used with the alumina posts.

The manufacturer's recommended heating prevention method, however, works only to prevent heating by electromagnetic induction and would not prevent antenna‐effect heating. An entirely different method would be required to prevent heating by the antenna effect. Fortunately, the GK headframe geometry is not a problem at 1.5 T or 3 T field strengths, because the dimensions are too small to heat by this method. However, if the static field operated at 7–10 T, the implant dimensions required for the antenna effect would be decreased to ~30−50 cm in air, and as small as just a few cm in the human body, depending on the type of tissue or organ where the implant is located, with the amount of heating dependent on the implant's depth below the surface and its angle with respect to the applied EM wave. This effect may be a major concern for patients with small implants in the human body, such as stents or aneurysm clips, that could pose a severe burn hazard at very high field strengths. Thus, with current MRI scanners operating at fields up to 7 T, the antenna effect may become the dominant heating hazard in the near future.

One of the key implications of this study is that therapists and other health professionals who image GK patients with headframes in MRI scanners and have not yet obtained insulated posts need to be aware of new safety regulations (such as the requirement to use only insulated posts for MRI scans of GK patients with headframes). It may be useful for GK health professionals to obtain additional training to understand how RF interacts with and potentially heats the human body through resistive heating in loops. It should be understood how current loops could potentially form through complex combinations of parts of a patient, a patient monitoring device, or instrument wiring via capacitive coupling, especially for higher field MRI units. The more complex heating mechanism of the antenna effect should also be understood by GK health professionals so that they are aware that thermal injuries can occur by mechanisms other than EM induction. Tables of conductivities and permittivities of different organs and tissues within the human body should be made readily available, along with dimensions of conducting implants that lead to antenna‐effect heating in various tissues in different field strength MRI scanner.

## V. CONCLUSIONS

This study shows that heating caused by the RF field of a 3T MRI scanner due to electromagnetic induction, as described by Faraday's law, occurs at the GK headframe screws when melon phantoms are mounted in the headframe during stereotactic MR imaging. Titanium screws combined with the uninsulated alumina posts result in maximum heating at the screw tips. This heating can be greatly reduced to negligible levels by the use of insulating nuts (the manufacturer's recommended procedure) that electronically separate the metallic screws from the posts. This method would, in principle, work for any conducting headframe post material (e.g., steel and carbon fiber).

There is increased risk of thermal injury at field strengths higher than 3T. The antenna effect was ruled out as the cause of headframe screw heating. However, above 3T it poses an increased risk of internal heating for GK and other patients with metallic implants having lengths suitable for standing wave formation (such as pacemaker wires), because resonant length decreases with increasing field strength and RF frequency. Thus, injury may be possible with even small‐dimension implants, such as aneurysm clips and stents. Electromagnetic induction, the principal heating mechanism identified for GK patients with attached headframes, also poses an increased risk because higher RF frequencies at field strengths above 3T may create unintended current paths through unintended capacitances. Thus, at sufficiently high magnetic fields beyond 3T, the electrically insulating nuts may no longer protect against induced heating. Their effective protection will need to be validated before use for GK patients with headframes undergoing stereotactic very high‐field (e.g., 4T, 7T, and higher) MR imaging.

## ACKNOWLEDGMENTS

The authors would like to thank S. Shave at General Electric Healthcare, O. Eriksson at Elekta, and Lisa Wilkins and Darrell Sloan at the Department of Radiation Oncology at Wake Forest University for their time and help with this project. This work is supported by the TRADONC post‐doctoral training program at Wake Forest University School of Medicine, funded by Grant No. NCI‐32 CA113267.
